# The short life of the volcanic island New Late’iki (Tonga) analyzed by multi-sensor remote sensing data

**DOI:** 10.1038/s41598-020-79261-7

**Published:** 2020-12-18

**Authors:** Simon Plank, Francesco Marchese, Nicola Genzano, Michael Nolde, Sandro Martinis

**Affiliations:** 1 German Aerospace Center (DLR), German Remote Sensing Data Center, 82234 Oberpfaffenhofen, Germany; 2 National Research Council of Italy (CNR), Institute of Methodologies for Environmental Analysis (IMAA), 85050 Tito Scalo, Italy; 3 School of Engineering, University of Basilicata, 85100 Potenza, Italy

**Keywords:** Natural hazards, Solid Earth sciences

## Abstract

Satellite-based Earth observation plays a key role for monitoring volcanoes, especially those which are located in remote areas and which very often are not observed by a terrestrial monitoring network. In our study we jointly analyzed data from thermal (Moderate Resolution Imaging Spectrometer MODIS and Visible Infrared Imaging Radiometer Suite VIIRS), optical (Operational Land Imager and Multispectral Instrument) and synthetic aperture radar (SAR) (Sentinel-1 and TerraSAR-X) satellite sensors to investigate the mid-October 2019 surtseyan eruption at Late’iki Volcano, located on the Tonga Volcanic Arc. During the eruption, the remains of an older volcanic island formed in 1995 collapsed and a new volcanic island, called New Late’iki was formed. After the 12 days long lasting eruption, we observed a rapid change of the island’s shape and size, and an erosion of this newly formed volcanic island, which was reclaimed by the ocean two months after the eruption ceased. This fast erosion of New Late’iki Island is in strong contrast to the over 25 years long survival of the volcanic island formed in 1995.

## Introduction

### Volcanic islands formed in recent decades

Eruptions of submarine volcanoes can form new islands. The material of which the new formed island is composed of determines whether it is short-lived or whether it can survive. Volcanic islands that last longer can be valuable for scientists to study the colonization of virgin land by plants and animals. The most famous volcanic island formed in the last decades is Surtsey, which appeared off the coast of Iceland in 1963. Other newly formed volcanic islands, which survived erosion of the ocean waves until today, are for example Nishinoshima and Niijima (formed in 1974 and in 2015 about 1000 km south of Tokyo) as well as Zubair (formed in 2013 off the coast of Yemen). In late 2014/early 2015, a surtseyan eruption formed a new island, Hunga Tonga-Hunga Ha'apai, which connected two older Tongan islands^1^. Newly formed islands that survive over a longer period can also expand a country’s offshore territorial rights. The UN Convention on the Law of the Sea allows countries to claim rights over shipping, mining and fishing up to 200 nautical miles (ca. 370 km) from their coast. Such a newly claimed island off a country’s coast can be used as the basis for a new offshore territorial claim, too. Other volcanic islands survive only a few months or years until their area is again reclaimed by the ocean. Examples are Kuwae (Vanuatu, erupted last in 1974), Fukutoku-Okanoba (erupted last in 1986 off the coast of Japan), Kavachi (Solomon Islands, erupted last in 2003), Metis Shoal and Home Reef (Tonga Islands, erupted last in 1995 and in 2006) as well as New Late’iki (Tonga Islands), whose formation and temporal evolution is the topic of this article.

### Regional setting and previous eruptions

**Figure 1:**
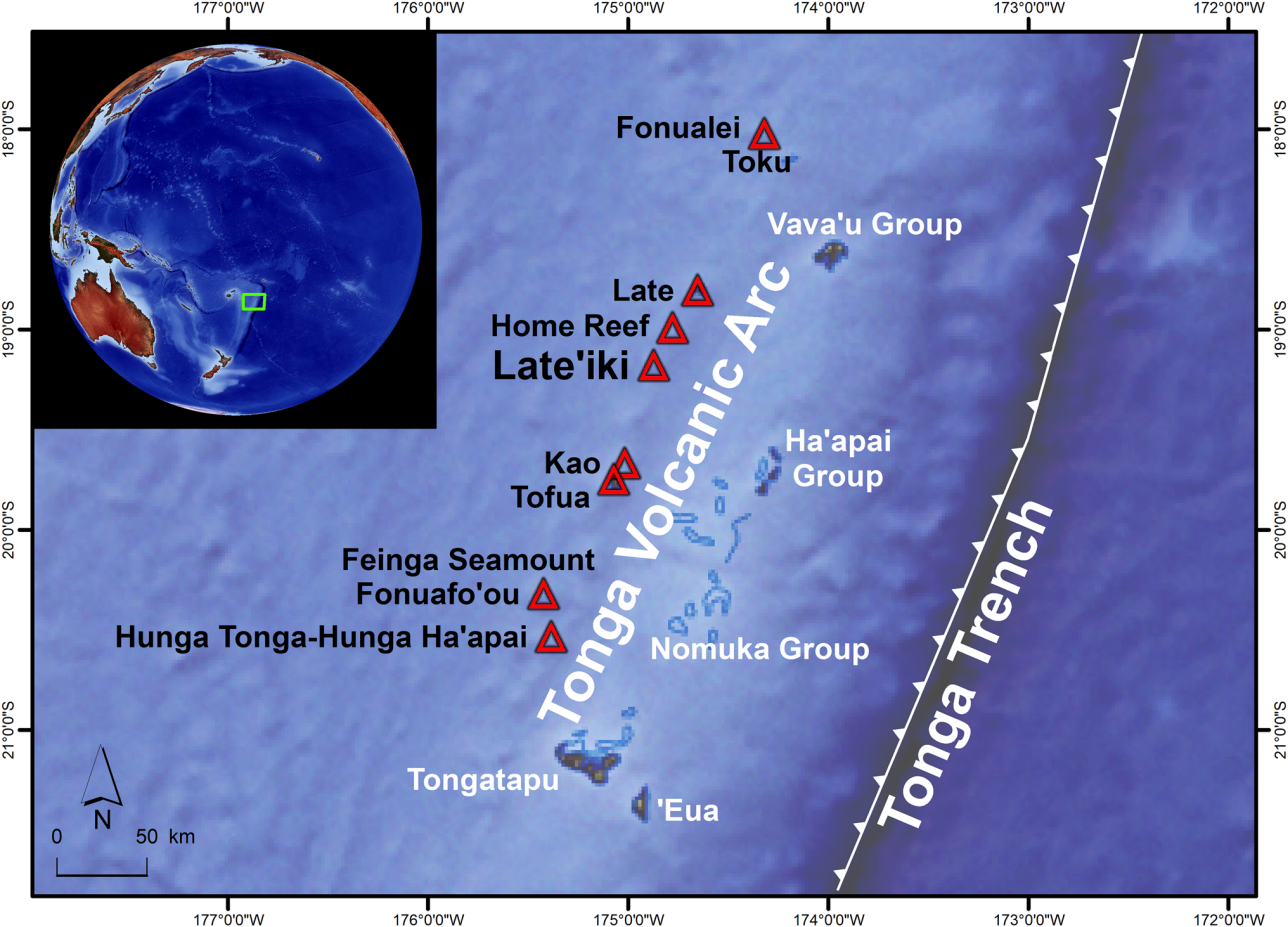
Late’iki is one of several submarine and island volcanoes (red triangles) located on the Tonga Volcanic Arc, west of the Tonga Trench in the South Pacific. Besides at Late’iki Volcano, surtseyan eruptions were also observed at Home Reef in 2006^46^ and at Hunga Ha'apai in 2009^59^ and in 2014/2015^1^. The green rectangle marks the location of the detailed map. Map changed after^4^ and^60^. Background: Made with Natural Earth (naturalearthdata.com).

Late’iki, formerly called Metis Shoal, is one of several submarine and island volcanoes on the western side of the Tonga Trench in the South Pacific. The Tongan Islands are a double island chain located at the northern end of an island arc system that extends discontinuously in a north–north-eastern direction from northern New Zealand to south of Samoa^2^). Late’iki (19.18°S, 174.87°W) is located on the Tonga Volcanic Arc about 56 km north of the Tongan island Kao and about 47 km south of the island Late (Fig. 1).

Late’iki Volcano has produced ephemeral islands multiple times. Eruptions at Late’iki Volcano were reported six times between 1851 and 1894^2^, in 1967–1968^3^, in 1979, in 1995 and in 2019. The volcanic islands built during the eruptions in 1967 and 1979 were composed of pyroclastic debris and survived for a few months only before eroding beneath the sea surface^4^. In 1995, an island was produced that survived over 25 years until an eruption in mid-October 2019, which is analyzed in detail in this article. Late’iki Volcano typically produces dacitic lava^2,5^.

The following gives a short description of the eruptions between 1967 and 1995 (summarized from reports of the Global Volcanism Program GVP^6^). On 12 December 1967, sailors observed an eruption at the location of Late’iki. This eruption, which lasted for about 1 month, formed a new volcanic island composed of dacitic tuff, which eroded beneath the sea level by the end of February 1968. A detailed description of the 1967/1968 eruption at Late’iki Volcano is given by^3^. During another eruption, which was observed from 19 June 1979 onwards, again an island was born, which, by mid-July 1979, was about 300 m long, 120 m wide, and 15 m high, as reported by geologists who visited the island. By early October 1979, this island was again eroded beneath the sea surface. 16 years later, on 6 June 1995, a new eruption was observed, which formed a new island. By late June 1995, when the dome growing stopped, the island had a size of about 300 m × 250 m and a height of ca. 50 m. Contrary to earlier eruptions at Late’iki Volcano, in 1995 the island was formed by a growing lava dome that was composed of hardened lava and not of tuff. Therefore, the island survived much longer than the ones produced during previous eruptions. It is assumed that the island that was observed at Late’iki Volcano during an airplane overflight in December 2006 is the same island that was produced during the eruption period in 1995^6^. We also observed this island in Landsat-7 scenes acquired in 1999, 2000, 2001 and 2003 as well as in a series of Sentinel-2 satellite imagery acquired between 2015 and 2019.

Here, we present a detailed multi-sensor satellite imagery-based analysis of the mid-October 2019 eruption at Late’iki Volcano and the temporal evolution of a newly formed island, called New Late’iki. This new eruption at Late’iki Volcano was first noted by a ship at 08:00 local time on 14 October 2019, and confirmed by the following morning by a pilot from Real Tonga Airlines, who reported a steam plume of 4.6–5.2 km altitude^6^.

### Remote sensing of volcanic eruptions

Many of the world’s volcanoes are located in remote areas and are not readily accessible, especially during explosive eruptions. It is therefore difficult to gain in situ information. Satellite-based Earth Observation (EO) provides extensive capabilities to support detection, monitoring and investigation of active volcanoes^7^.

Satellite-based volcano monitoring is often performed on data from thermal, optical and synthetic aperture radar (SAR) sensors. Thermal EO has been a well-established volcano monitoring technique since the early 1980s, beginning with the NASA Landsat Thematic Mapper (TM) series and the Advanced Very High Resolution Radiometer (AVHRR) on-board the National Oceanic and Atmospheric Administration (NOAA) satellites, e.g.^8^. Thermal satellite imagery has been used to investigate a variety of thermal volcanogenic emitting phenomena, such as active lava flows (e.g.^9–11^), lava lakes (e.g.^12^), fumarolic fields (e.g.^13,14^), and lava domes (e.g.^15^). The high temporal resolution data from geostationary satellites such as the Geostationary Operational Environmental Satellite (GOES), Meteosat (with the Spinning Enhanced Visible and InfraRed Imager SEVIRI sensor) and Multifunctional Transport Satellites (MTSAT) were used by^16,17^ as well as by^18,19^ (HOTSAT system)^20^, (HOTVOLC system) and^21^ (RST_VOLC_) among others, to analyse volcanic activity in near real-time.

Important developments in automated thermal hotspot detection approaches are based on the Moderate Resolution Imaging Spectrometer (MODIS) provided by the Middle InfraRed Observation of Volcanic Activity (MIROVA) system^22^ and by MODVOLC^23^. The high capabilities for thermal anomaly detection for the Visible Infrared Imaging Radiometer Suite (VIIRS) sensor were demonstrated by^24,25^. In^26^ thermal data from MODIS and VIIRS were combined to estimate lava discharge rates. Moreover, hotspot detection algorithms have also been developed for processing data from the Advanced Spaceborne Thermal Emission and Reflection Radiometer (ASTER) (e.g.^27^). A detailed analysis of thermal volcanic activity using high-resolution thermal data from the FireBIRD small experimental satellite mission were demonstrated by^28^. In^29^, based on shortwave infrared (SWIR) and thermal infrared (TIR) Landsat-8 data, the so-called thermal eruption index (TEI) was proposed to enable a differentiation of thermal domains within a lava flow.

High-resolution optical satellite imagery is ideally suited for the detailed analysis of, for example, lava flows and pyroclastic density currents (e.g.^18,25,30,31^).

SAR sensors provide useful imagery at day and night and almost completely independent of the weather. With SAR data, it is possible to observe the surface of a volcano also during explosive eruption events when the visibility and applicability of optical sensors are limited by clouds. Numerous studies have investigated SAR data for volcano monitoring, based on either SAR amplitude information (e.g.^32–36^) or SAR interferometric processing of both amplitude and phase information (e.g.^37–39^). Interferometric processing is suitable for monitoring terrains free of vegetation and snow, which deform only slowly. The advantage of SAR amplitude data is that also information about the volcanic surface can be obtained when major changes occur, e.g. due to explosive eruptions^40^.

A joint analysis of data from thermal, optical and SAR sensors can make a significant contribution to the understanding of volcanic processes^7^. Examples for multi-sensor satellite analysis of active volcanoes are the studies of^41^, who analyzed optical and SAR data to investigate the Puyehue-Cordon Caulle eruption in 2011, and of^42^, who combined thermal remote sensing and differential interferometric SAR analysis to study the Nabro Volcano eruption in 2011. A joint analysis of SAR, optical and thermal imagery was performed by^43^ to investigate the rapid growth and tsunami-genic collapse of a littoral lava dome at Kadovar Volcano, Papua New Guinea in 2018.

## Results

### Multispectral satellite remote sensing monitoring

**Figure 2:**
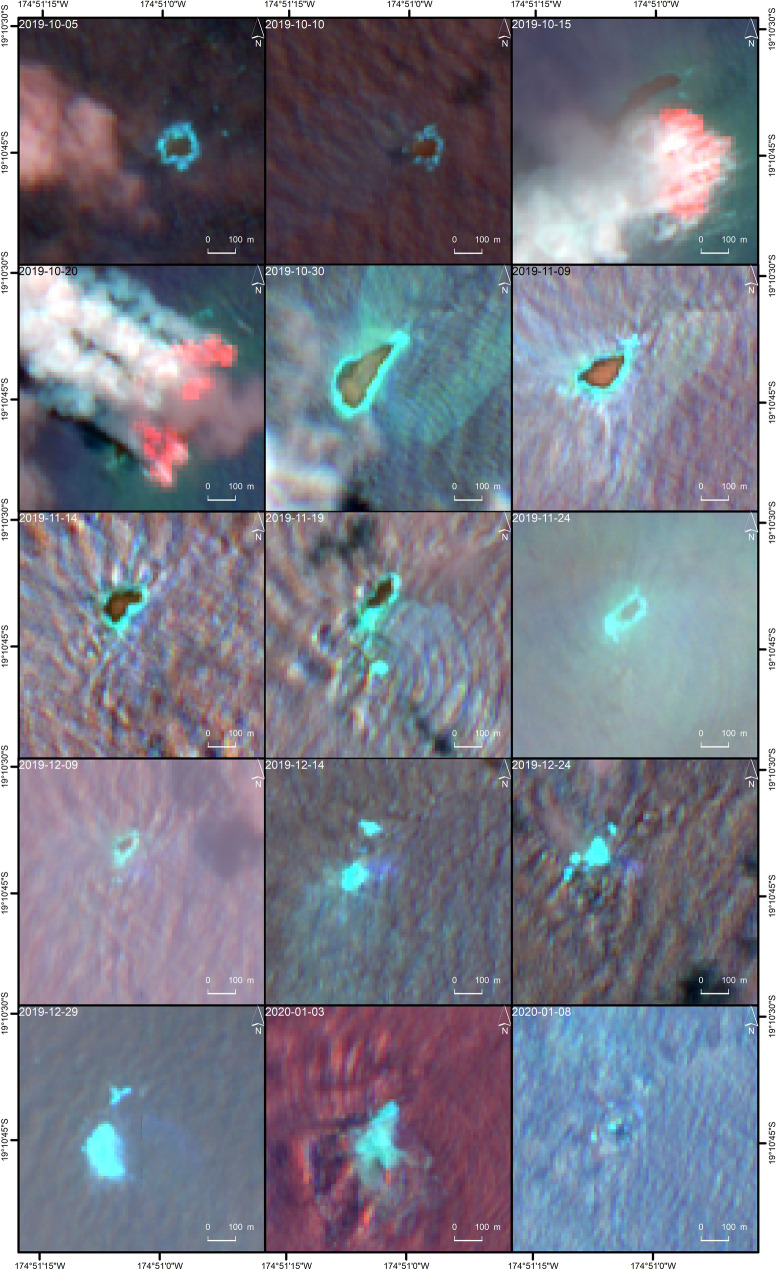
Time series of Sentinel-2 imagery (band combination 12/4/2) over Late’iki Volcano from 5 October 2019 until 8 January 2020. The old Late’iki Island is clearly visible until finally 10 October 2019. The acquisitions of 15 and 20 October 2019 show the ongoing eruption and the birth of New Late’iki Island, which then eroded during the next two months. Background: Sentinel-2 Copernicus data (2019).

In order to get a first overview of the temporal evolution of Late’iki Volcano in autumn 2019, we analyzed high spatial resolution multispectral data. Figure 2 shows a time series of Sentinel-2 imagery with the band combination 12/4/2 (short wave infrared (SWIR), center wavelength λ = 2.190 µm/red λ = 0.665 µm/blue λ = 0.490 µm). At the beginning of this time series, we clearly see a rectangular shaped oceanic island with an E-W extension of ca. 80 m and an N-S extension of ca. 50 m. A ring of shallower water (shown by the cyan color) surrounds the island. The situation at Late’iki Volcano was stable until finally 10 October 2019.

On the next available Sentinel-2 scene, the situation changed completely: The Sentinel-2 acquisitions of 15 and 20 October 2019 show strong intense reflectance in the SWIR (red signature) around the former location of Late’iki Island. This strong SWIR signature and the large emission of water vapor show an ongoing eruption at Late’iki Volcano. Furthermore, on the 15 October 2019 scene, a new island located to the NW of the position of Late’iki Island became visible. This new island, called New Late’iki in the following, had an NE-SW extension of ca. 350 m and an NW–SE extension of maximum ca. 60 m on 15 October 2019.

**Figure 3:**
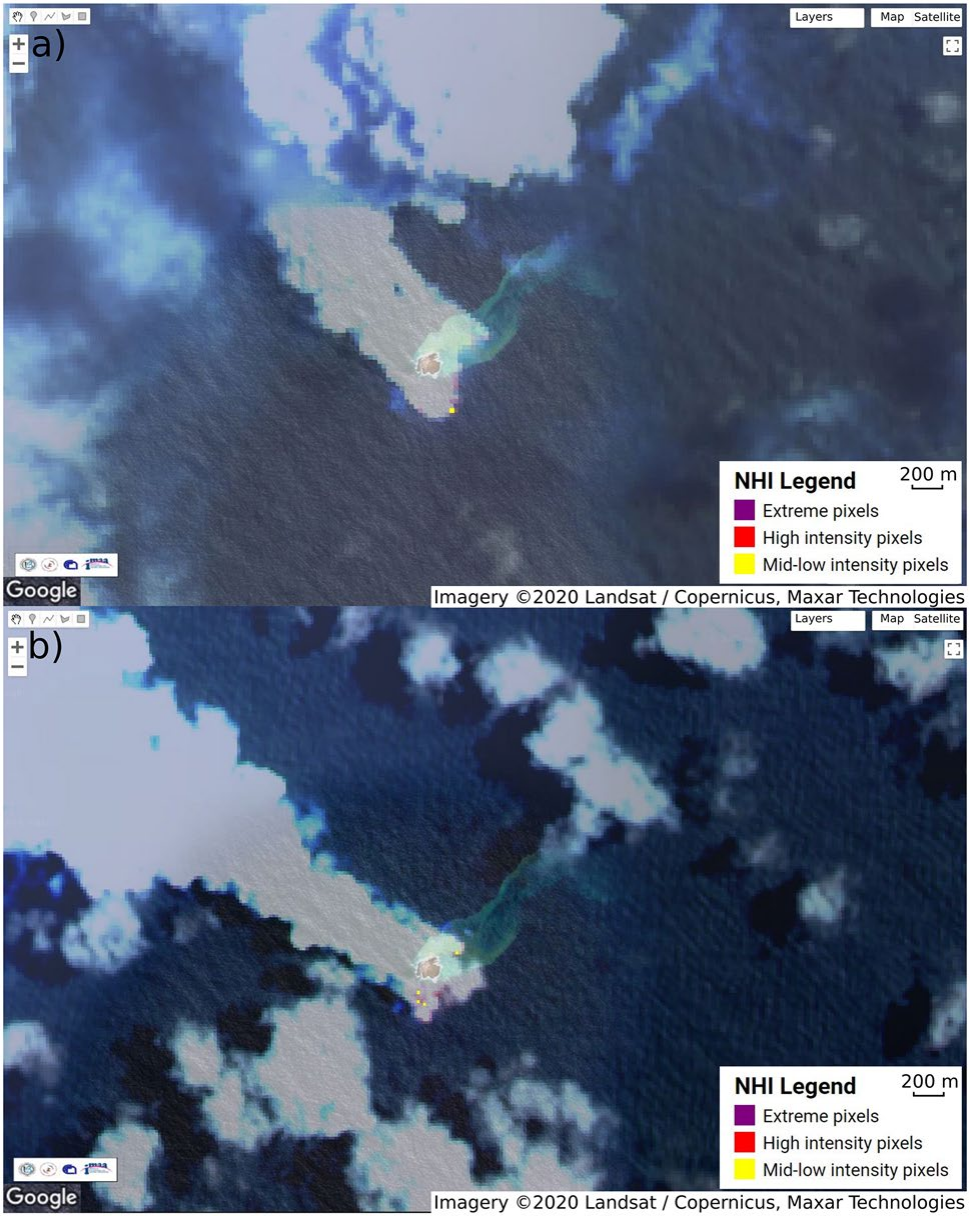
Volcanic hotspot of mid-low intensity identified at Late’iki Volcano by the NHI tool in (**a**) Landsat-8 OLI data on 16 October 2019 and (**b**) in Sentinel-2 MSI data on 20 October 2019. Clouds and volcanic plume are shown in transparent grey color. Background: Google Earth satellite image showing old Late’iki Island prior to the mid-October 2019 eruption. Imagery Landsat/Copernicus (2020), Maxar Technologies.

Application of the Normalized Hotspot Indices (NHI) tool (cf. “Multispectral satellite remote sensing monitoring”^44,45^) on Landsat-8 Operational Land Imager (OLI) and Sentinel-2 Multispectral Instrument (MSI) data resulted in the identification of a thermal anomaly on 16 October 2019 (Landsat-8, see the single hotspot pixel in Fig. 3a), appearing more extended on 20 October 2019 (Sentinel-2, see hotspot pixels in Fig. 3b). Thereby, on the 16 October 2019 Landsat-8 OLI scene, the radiance over the thermal anomaly was 15.96 or 17.28 W × m^−2^ × sr^−1^ × µm^−1^ in the 1.6 or 2.2 µm wave band, respectively. In the 20 October 2019 Sentinel-2 scene, the total radiance over the four thermal anomalies was 53.815 or 61.167 W × m^−2^ × sr^−1^ × µm^−1^ in the 1.6 or 2.2 µm wave band, respectively. Thereby, a hotspot area of ca. 900 m^2^ and 1,600 m^2^ were detected by the NHI tool in the aforementioned Landsat-8 (16 October 2019) and Sentinel-2 (20 October 2019) acquisitions. These values provide a rough estimate of the total hotspot area also because of the plume affecting the number of detected hotspot pixels (see Fig. 3).

On the Sentinel-2 MSI scene of 15 October 2019, the NHI tool did not detect any thermal anomaly possibly due to plume-contaminated pixels (cf. also Fig. 2). Nevertheless, for this Sentinel-2 image offline analysis of the NHI results performed using the Sentinel Application Platform (SNAP) tool from the European Space Agency (ESA) showed a clear increase of the NHI_SWIR_ index (cf. “Multispectral satellite remote sensing monitoring”) over plume/hotspot pixels, whose plot is not shown here.

**Figure 4:**
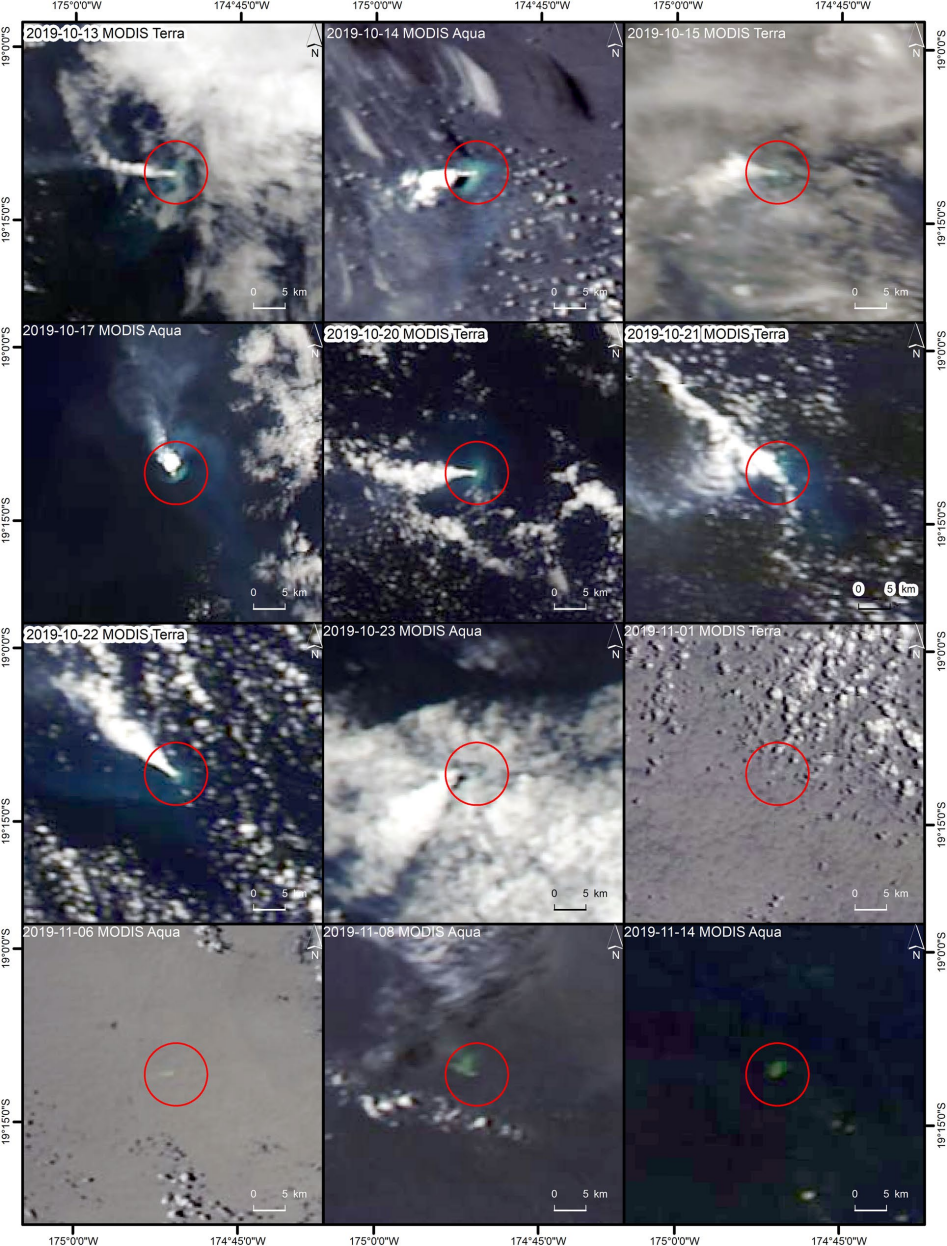
Volcanic water vapor plumes over Late’iki Volcano (red circle) as observed by MODIS Aqua and Terra from 13 to 23 October 2019. The imagery from 1 to 14 November 2019 show the temporal evolution of New Late’iki Island as observed by the MODIS sensor. MODIS RGB imagery with band combination 1/4/3. Background: NASA Worldview (2019).

The low spatial, but high temporal resolution observation by MODIS and VIIRS showed volcanic activity at Late’iki Volcano from 13 until finally 23 October 2019. Figure 4 shows some example imagery of MODIS in red/green/blue (RGB) band combination 1/4/3 (cf. “High frequent & low spatial resolution satellite monitoring”).

**Figure 5:**
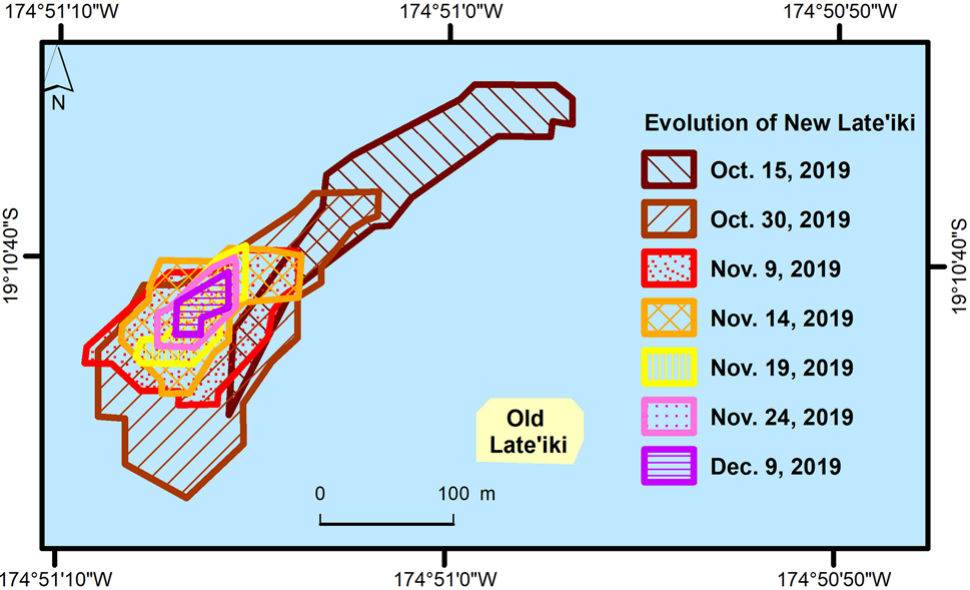
The spatio-temporal evolution of New Late’iki Island as mapped from Sentinel-2 imagery shown in Fig. 2.

New Late’iki was covered by the water vapor emissions on the 20 October 2019 Sentinel-2 image. The following Sentinel-2 scenes from 30 October 2019 onwards (the 25 October 2019 image was completely cloudy over the area of interest AOI) show that the shape and size of this newly born island dramatically changed over time. New Late’iki was eroded during the next six weeks. Figure 5 shows the spatio-temporal evolution of New Late’iki mapped using the Sentinel-2 time series shown Fig. 2. From 14 December 2019 onwards, the island was completely covered by the sea. From 17 February 2020 onwards, the remains of the former island were hardly visible in the high-resolution Sentinel-2 imagery. Only discoloration of the sea water around the former location of the island is still visible in Sentinel-2 imagery acquired at the time writing this manuscript (November 2020).

### SAR remote sensing satellite monitoring

**Figure 6:**
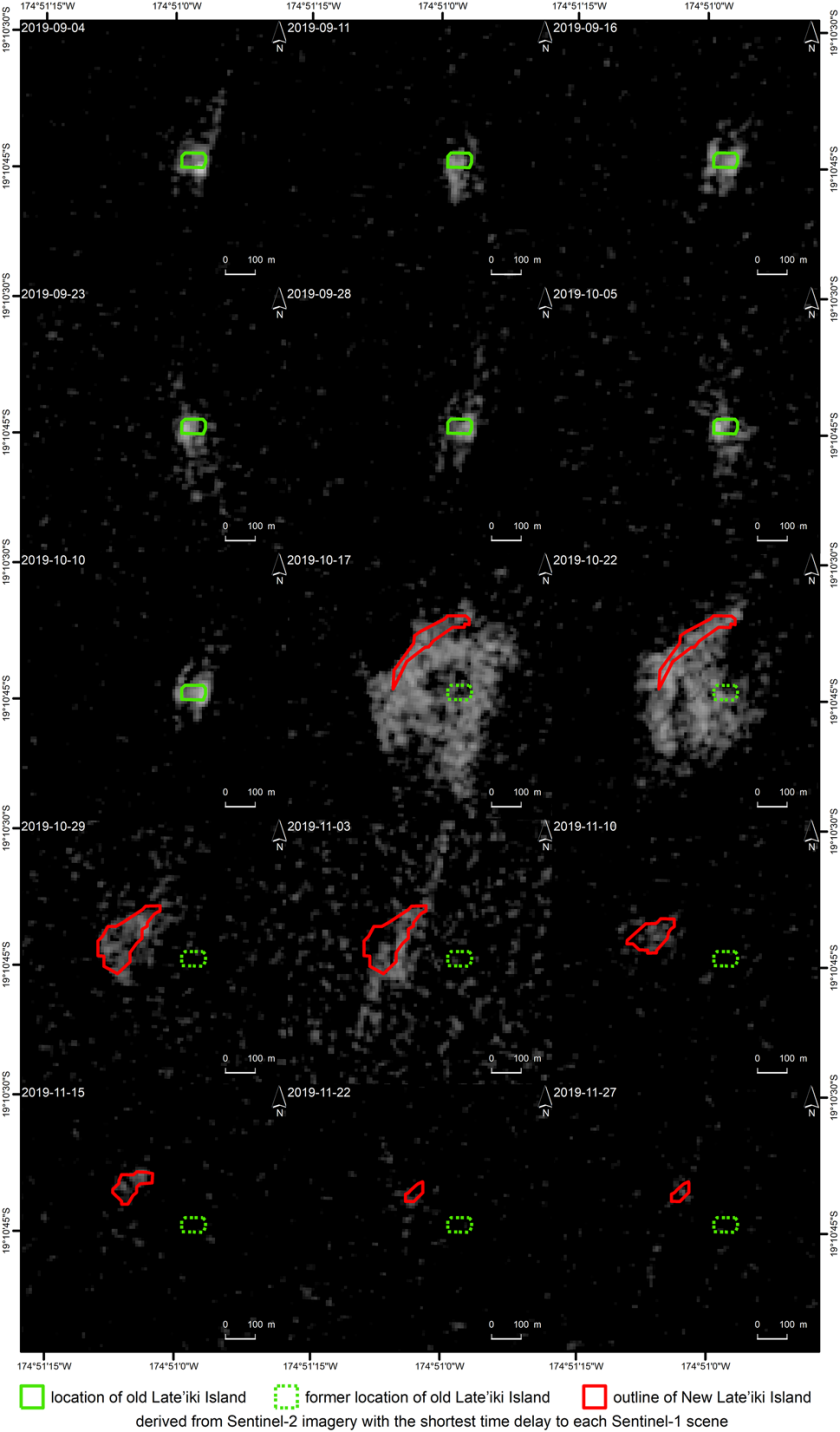
Late’iki Volcano as observed by Sentinel-1 SAR VH-polarized imagery from 4 September 2019 to 27 November 2019. Background: Sentinel-1 Copernicus data (2019).

Figure 6 (and Supplementary Fig. S1) show the temporal evolution of the Sentinel-1 SAR backscatter of the vertical/horizontal (VH) and the vertical/vertical (VV) polarization over the AOI. From the beginning of the time series on 4 September 2019 until finally 10 October 2019, higher backscatter at the location of the old Late’iki Island is visible, especially in the VH polarization data series. This backscatter signalizes the location of the old Late’iki Island. The VV polarization is more influenced by the sea surface roughness (waves) than the VH polarization. On the 17 October and on the 22 October 2019, Sentinel-1 scenes increased backscatter of the SAR signal is visible over a larger area around the old Late’iki Island. This area has a diameter of ca. 700 m (N-S) and 500 m (E-W). New Late’iki Island is located at the NW edge of the area with stronger backscatter. This area became smaller in E-W direction on 22 October 2019.

The following Sentinel-1 acquisitions (from 29 October 2019 onwards) confirm the observation of the multispectral data (Sentinel-2): The area of New Late’iki Island continuously decreased. A last signature of New Late’iki is visible on the 27 November 2019 Sentinel-1 SAR image.

**Figure 7:**
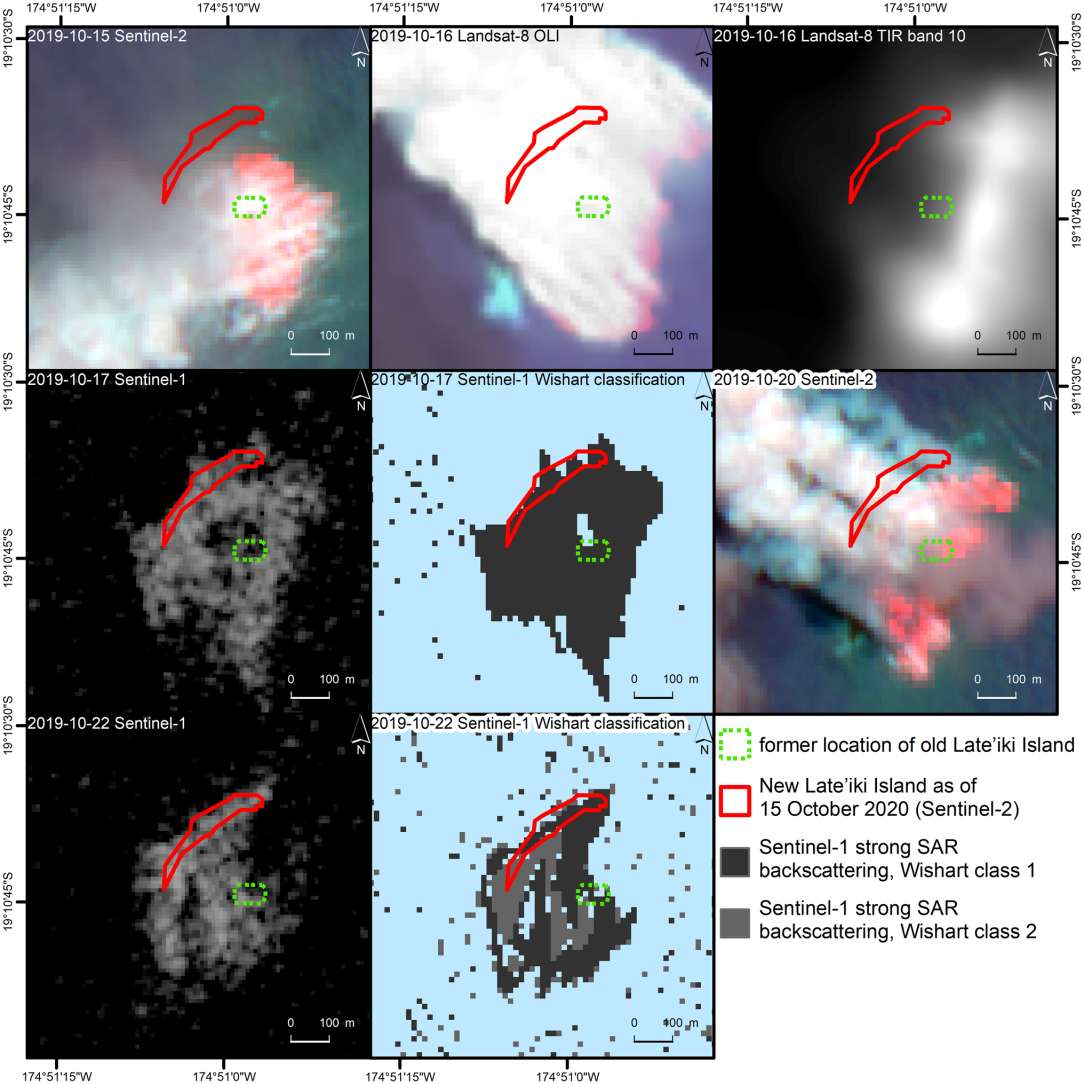
Surtseyan eruption at Late’iki Volcano monitored by Sentinel-2 (15 and 20 October 2019), Landsat-8 (16 October 2019) and Sentinel-1, including result of the polarimetric Wishart classification (17 and 22 October 2019). Background: Sentinel-1 and Sentinel-2 Copernicus data (2019). Landsat-8 image courtesy of the U.S. Geological Survey.

Besides the visible analysis of the SAR backscatter, also a more detailed investigation of the polarimetric signatures was performed for the two Sentinel-1 scenes showing the increased SAR backscatter around old Late’iki Island. The Sentinel-1 acquisitions of 17 and 22 October 2019 were acquired during the ongoing eruption at Late’iki Volcano. Figure 7 shows the results of the polarimetric Wishart unsupervised classification, which is described in “Materials and methods”. The Wishart classification clearly separated the area of strong backscatter from the surrounding calmer open water ocean area shown in the Fig. 6 on 17 and 22 October 2019. Thereby, on the 17 October 2019 scene, the strong backscattering area was classified as one common class, while it was classified as two separate classes on the 22 October 2019 image.

The two high spatial resolution TerraSAR-X SpotLight (SL) and HighResolution SpotLight (HS) scenes were acquired shortly before New Late’iki Island was completely eroded and covered by the sea. On 2 December 2019, there was a backscattering signal visible at the location New Late’iki. The identification of the island two days later is hardly possible (Supplementary Fig. S2).

### Joint analysis of all remote sensing data

**Figure 8:**
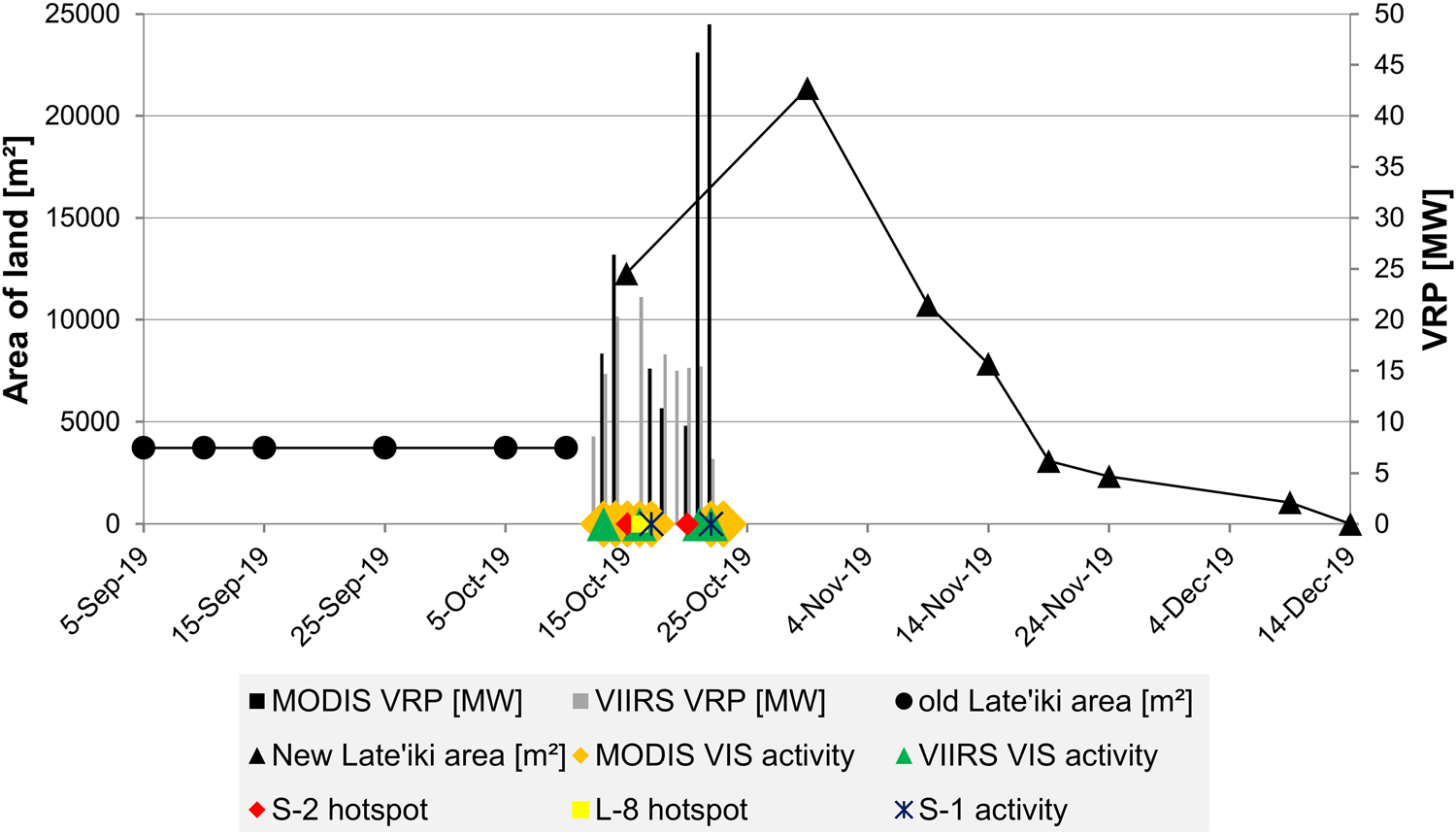
Joint analysis of (1) the spatio-temporal evolution of old Late’iki Island and New Late’iki Island as derived from Sentinel-2 imagery; (2) the VRP detected by MODIS and VIIRS during the eruption; (3) volcanic hotspots detected by Sentinel-2 and Landsat-8 using the NHI tool; (4) visible (VIS) volcanic activity as observed by MODIS and VIIRS RGB imagery as well as in Sentinel-1 SAR data.

Figure 7 shows the ongoing eruption at Late’iki Volcano in mid-October 2019 monitored by Sentinel-1, Sentinel-2 and Landsat-8. The area of strong SWIR signature (red, Sentinel-2 and Landsat-8) corresponds well with the area of strong SAR backscattering (Sentinel-1), with the advantage of the SAR data providing information of the AOI independent of the any masking by the volcanic plume. Strong thermal signatures are only visible for the area not covered by the plume (see Fig. 7, Landsat-8 OLI and TIR). The area of high thermal signatures covers the hotspot detected by the NHI tool (cf. Fig. 3), but also a larger region around the eastern edge of the plume. The volcanic water vapor plume also causes some blurring of the thermal signature. Figure 8 summarizes all observations from the multi-sensor remote sensing analysis of this eruption. The figure shows the spatio-temporal evolution of the Late’iki Island, which was destroyed during the eruption, and of the newly born New Late’iki Island. This island reached its maximum area on 30 October 2019 (ca. 21,000 m^2^). Then, New Late’iki Island was eroded during the next six weeks with an average erosion rate of ca. 464 m^2^ per day. Thereby, we observed from 30 October until 19 November 2019 an erosion rate of ca. 960 m^2^ per day. After 19 November 2019 the erosion rate slowed down to ca. 124 m^2^ per day. From 14 December 2019 onwards, New Late’iki Island was completely covered by the sea.

The first signature of a volcanic activity at the area of Late’iki Volcano was detected by VIIRS on 12 October 2019. As the thermal sensors analyzed in this study, MODIS and VIIRS perform several overflights per day, the volcanic radiant power (VRP) of the overflight with the highest VRP value within each day was considered in the analysis. Figure 8 shows increasing VRP during the first three days of the eruption with a maximum of 26.4 MW detected by MODIS. The average VRP over the next six days was ca. 15 MW. Then, on 21 and 22 October 2019, the last thermal hotspots were detected by MODIS and VIIRS with much stronger VRP values of up to 49 MW. On 23 October 2019, the visual analysis of the MODIS RGB channels showed the last volcanic activity at Late’iki Volcano during this eruption period.

The analysis of the higher spatial resolution imagery (hotspot detection based on Sentinel-2 MSI and Landsat-8 OLI as well as analysis of Sentinel-1 SAR data) showed volcanic activity from 15 October until finally 22 October 2019, which perfectly matches with the lower resolution but more frequent observations from MODIS and VIIRS (Fig. 8).

Due to the long distance of the seismic stations to Late’iki Volcano—the closest seismic station is located on the Fiji Islands, at a distance of around 770 km away from Late’iki Volcano—and because of other seismic events (earthquakes), it was not possible to detect clear evidence of seismic readings of Late’iki Volcano unrest. Supplementary Fig. S3 shows the cumulative number of earthquakes for an area of 100 km radius around Late’iki Volcano and with a depth of < 100 km.

## Discussion

According to our knowledge, there are no other scientific publications about the mid-October 2019 eruption at Late’iki Volcano and the formation of New Late’iki Island. This article presents a joint analysis of thermal, optical and SAR satellite data, which enabled us to get a comprehensive picture of this volcanic eruption.

SAR data has the advantage of providing useful information at day and night, independent of the cloud coverage over the area of interest. However, it is more complicated to interpret these SAR imageries due to the influence of breaking waves at the coast—especially at small islands. Prior to the mid-October 2019 eruption at Late’iki Volcano, the old Late’iki Island is especially well visible in the VH polarized Sentinel-1 SAR images. This cross-polarization is much better suited for object identification within the ocean. For instance, also for SAR-based ship detection, cross-polarized SAR data are preferred. In contrast to this, there is a much higher influence of the sea surface roughness in VV polarized SAR data, which makes the identification of objects (small islands or ships) much more complicated.

Figure 7 shows the ongoing eruption at Late’iki Volcano in mid-October 2019 as observed by SAR (Sentinel-1), optical (Sentinel-2 and Landsat-8 OLI) and thermal (Landsat-8 TIR) remote sensing sensors. We interpret the area of strong SWIR signature (red, Sentinel-2 and Landsat-8 OLI), of strong thermal signature (Landsat-8 TIR) and of strong SAR backscattering (Sentinel-1), respectively, as the area affected by the ongoing surtseyan eruption. This eruption took place in shallow waters around old Late’iki Island. The water in this area was bubbling and partly boiling during the ongoing eruption. At some regions of the eruption site, new land grew above sea level, which then formed New Late’iki Island. The size of the area affected by strong SAR backscattering as well as the characteristics of the backscattered signal changed during the ongoing eruption as it is shown by the different Wishart classification results for the Sentinel-1 images (Fig. 7).

The automatic MODVOLC thermal alert system^23^ recorded three thermal alerts from Late’iki, one each on 18, 20, and 22 October 2019. These thermal hotspot detections match well with our observations (cf. Fig. 8). They included the identification of thermal anomalies on both Landsat-8 OLI and Sentinel-2 MSI data with the NHI tool.

Our high frequent monitoring of the mid-October 2019 Late’iki Volcano eruption showed emission of water vapor (steam plume), but no volcanic ash was visible (cf. Figs. 2 and 4). This is in line with the aviation advisory reports of the Wellington VAAC issued on 14 and 22 October 2020, which described a steam plume reported by pilots at altitudes up to 4.6–5.2 km or 3.7 km, respectively. No ash plume was reported^6^.

The estimates of the total radiance values over the thermal anomalies detected by Landsat-8 (16 October 2019) and by Sentinel-2 (20 October 2019) have to be considered with some caution due to the influence of solar irradiation during day time (cf. “Multispectral satellite remote sensing monitoring”). However, there were no night time acquisitions of this eruption available.

Analysis of sulphur dioxide (SO_2_) data from the Sentinel-5P mission by means of the Google Earth Engine (GEE) showed no intensified SO_2_ emission in the surroundings of Late’iki Volcano. However, the discoloration of sea water visible around Late’iki during the ongoing eruption, for example in the Sentinel-2 imagery acquired 20 October 2019, but also after the eruption had ceased, for example in the Sentinel-2 images of 30 October 2019, 19 November 2019 and 14 December 2019 (Fig. 2). These independent satellite observations signalize that Late’iki Volcano may had been still emitting gas such as SO_2_, carbon dioxide (CO_2_) and hydrogen sulfide (H_2_S), as it was also observed during the 2006 eruption at Home Reef, Tonga^46^. These gas emissions seem to continue over a period of at least one year, as this discoloration around the former location of New Late’iki Island is still visible until the time writing this manuscript (November 2020).

The 1995 eruption at Late’iki Volcano formed a volcanic island composed of hardened lava^6^. This island survived over 25 years until the mid-October 2019 eruption when it disappeared. We assume that the remains of the 1995 born island collapsed during the 2019 eruption event. As described in “Regional setting and previous eruptions”, by late June 1995, the sub-aerial part of the formed lava dome had a size of about 300 m × 250 m (= 75,000 m^2^) and a height of ca. 50 m. As described in “Discussion”, we measured for this island in Sentinel-2 imagery, acquired shortly before the mid-October 2019 eruption, a size of ca. 80 m (E-W) × 50 m (N-S), i.e. an area of ca. 400 m^2^. Consequently, we can calculate an averaged erosion rate for the island formed in June 1995 old Late’iki of ca. 8.4 m^2^ per day, which is about 55-times lower than the erosion rate of New Late’iki Island (in average ca. 464 m^2^ per day). “Results” showed that the newly formed volcanic island, New Late’iki, was eroded within a relatively short time period of about two months only. Therefore, we assume that New Late’iki Island, formed in mid-October 2019, was composed of easily erodible material such as pyroclastic debris, as it was the case in the previous eruptions in 1967–68 as well as in 1979, when the newly formed islands at Late’iki Volcano survived for a few months only, too^3,4^.

In addition to the satellite data presented in the previous sections, we also analyzed other freely available satellite imagery. First, data from the geostationary satellites GOES-West and Himawari-8/-9 (with the sensor Advanced Himawari Imager AHI) were investigated. However, as the Tonga Islands are located at the very edge of both of these geostationary footprints, the spatial resolution in this area is very low (e.g., ca. 10 km for Himawari). Therefore, no sign of volcanic activity (neither by visual analysis of the data nor by thermal hotspot detection) could be detected with these data during the activity period of Late’iki Volcano. Second, the few available images of the ASTER sensor all showed a complete cloud coverage of the area of interest.

In a previous study, we showed that multi-platform satellite systems might contribute in characterizing eruptive activity in areas well monitored by traditional surveillance systems (see^25^). This contribution is even more important for monitoring of volcanoes which are located in remote areas, such as Late’iki Volcano and many others around the globe (e.g. in Oceania, the polar regions, Latin America, Siberia, etc.). For many of those volcanoes, there exists no seismic monitoring network. Even the closest seismic station in many cases is in far distances of several hundred or more kilometers. For instance, the closest seismic station is located in a distance of around 770 km away from Late’iki Volcano. At such long distances, only relatively large seismic signals can be recorded with a signal-to-noise ratio sufficient to enable a reliable source location to be inferred. The repeat cycle of Landsat is with 16 days too long for a detailed monitoring, especially due to possible cloud coverage of the scenes. After the failure of the Landsat-7 Scan Line Corrector on 31 May 2003, the monitoring of small volcanic island became even more difficult. Beginning in 2014/2015, after the launch of the first systematically acquiring high spatial and temporal resolution satellite sensors, Sentinel-1 (SAR) and Sentinel-2 (optical), monitoring of active volcanoes all around the globe and the analysis of their eruption events became more and more possible at a level of detail that was not possible in the past. The possibility for a combined monitoring by high resolution SAR (Sentinel-1) and optical sensors (Sentinel-2 combined with Landsat-8) is a great step forward as with SAR data we get useful information independent of the weather situation, which can then be even better interpreted as soon as the next clear sky optical image becomes available.

In general, a global monitoring of volcanoes, also of those located in remote areas, is important, as larger volcanic eruptions may have impacts not only on the direct surroundings of the active volcano, but also on distant regions. For example, collapses of volcanic islands may cause tsunami waves that could endanger inhabited coast lines at far distances. Additionally, large ash emissions during a volcanic eruption may strongly influence the regional air traffic.

## Conclusions

This article presented the multi-sensor Earth Observation (MODIS, VIIRS, Sentinel-2, Landsat-8, Sentinel-1 and TerraSAR-X) data-based analysis of the mid-October 2019 surtseyan eruption at Late’iki Volcano, located on the Tonga Volcanic Arc. During this recent eruption, the remains of an older volcanic island, which was formed in 1995, collapsed and a new volcanic island, called New Late’iki was formed. In contrast to its precursor island, which was composed of hardened lava and which survived over 25 years, this newly formed island New Late’iki was eroded within two months only, after the eruption ceased. We observed an erosion rate 55-times higher than the one of the island born in 1995. This fast erosion of New Late’iki indicates that the island formed by the mid-October 2019 eruption was composed of easily erodible material such as pyroclastic debris, i.e. the same material of which the short-living (few months) volcanic islands formed during the previous eruptions in 1967–68 and in 1979 were composed of. These results confirm the important role of multi-platform satellite observing systems in monitoring active volcanoes located in remote areas that very often are not observed by a terrestrial monitoring network (e.g. seismic stations), and for which satellite remote sensing may represent the unique source of information.

## Materials and methods

### Data

The following satellite data, acquired over Late’iki Volcano, were analyzed (Supplementary Table 1). In addition, we also consider seismic data available from distant stations as no local monitoring network was established on Late’iki or the neighboring Tonga islands.

#### High spatial resolution multispectral satellite monitoring

24 high-resolution (HR) optical Sentinel-2 datasets (from 5 September 2019 to 17 February 2020) and one Landsat-8 image were considered. Due to cloud coverage no further data from Sentinel-2 and Landsat-8 were useful. The repetition rate of Sentinel-2 is 5 days from the combined constellation of Sentinel-2A and B, and 16 days for Landsat-8. We investigated atmospherically corrected L2A and L1C data from the Sentinel-2 MSI with spatial resolutions of 10–20 m and from the Landsat-8 OLI (30 m spatial resolution).

#### High frequent & low spatial resolution satellite monitoring

All available acquisitions of MODIS and VIIRS from September 1, 2019 until the end of our observation period in February 29, 2020 were analyzed. VIIRS flying aboard the NOAA-20 and the Suomi National Polar-Orbiting Partnership (Suomi NPP) has a revisit time over the AOI of at least four acquisitions per day. MODIS flies on-board the NASA’s satellites Aqua and Terra and has a joint revisit time over Late’iki Volcano of four times per day. For visual inspection, the MODIS 250 m spatial resolution band 1 (red center wavelength λ = 645 µm) and the two 500 m spatial resolution bands 3 (blue λ = 469) and 4 (green λ = 555 µm) were used. For the visual inspection of the VIIRS data, the 375 m spatial resolution band I1 (red λ = 0.64 µm) and the 750 m spatial resolution bands M3 (blue λ = 0.483 µm) and M4 (green λ = 0.555 µm) were used. The MODIS and VIIRS data used for the visual inspection are corrected reflectance data that were derived from^47^. The surface reflectance data with a complete atmospheric correction (such as e.g. the MOD09 product) is only available over land surfaces and not for the area of Late’iki Volcano.

In addition, daily hotspot data from the thermal sensors MODIS and VIIRS were analyzed^48–50^. For the thermal hotspot detection, the 1 km spatial resolution MODIS mid-infrared (MIR) bands 21/22 (λ = 3.959 µm) and the thermal infrared (TIR) band 31 (λ = 11.03 µm) as well as the 375 m resolution VIIRS MIR band I4 (λ = 3.74 µm) and the TIR band I5 (λ = 11.45 µm) were used.

#### SAR remote sensing satellite monitoring

One Spotlight (SL; 1.7 m spatial resolution) as well as one HighResolution Spotlight (HS; 1.2 m spatial resolution) TerraSAR-X dataset were acquired in X-band (λ = 3.1 cm) with HH polarization over Late’iki. Furthermore, 15 C-band (λ = 5.5 cm) Sentinel-1 dual-pol (VV/VH) SAR images were analyzed (cf. Supplementary Table 1).

#### Seismic data

Data from the global seismic catalogs (Global Centroid-Moment-Tensor (CMT)^51^, Geophone^52^, USGS^53^) were investigated.

### Methods

#### Multispectral satellite remote sensing monitoring

As a first step, a visual analysis of the multispectral data of the sensors MODIS, VIIRS, Sentinel-2 and Landsat-8 was performed regarding detectable volcanic activity: (1) volcanic ash and water vapor plumes and/or (2) thermal signatures in the SWIR channels, respectively. For this visual inspection daytime imagery only were considered. For MODIS the band combination 1/4/3 was used, while the data of the bands 3 and 4 were sharpened to the 250 m spatial resolution using band 1. For VIIRS the band combination I1/M4/M3 was used with data of the two last bands sharpened to 375 m spatial resolution using band I1. The visual inspection of Sentinel-2 MSI data was performed using the band combination 12/4/2 (SWIR λ = 2.190 µm/red λ = 0.665 µm/blue λ = 0.490 µm). Landsat-8 OLI data were investigated using the band combination 7/4/2 (SWIR-2 λ = 2.20 µm/red λ = 0.665 µm/blue λ = 0.480 µm).

Furthermore, for Sentinel-2 MSI (L1C data) and Landsat-8 OLI (L1 data) detection of thermal volcanic anomalies and relative analysis we used the NHI algorithm described in detail by^44^. The NHI algorithm combines two normalized indices (Eqs. 1–2), analyzing the near infrared (NIR) and the SWIR radiances, to identify thermal anomalies in daylight conditions.

1 $$NHI_{SWIR} = \frac{{L_{2.2} - L_{1.6} }}{{L_{2.2} + L_{1.6} }}$$

2 $$NHI_{SWNIR} = \frac{{L_{1.6} - L_{0.8} }}{{L_{1.6} + L_{0.8} }}$$

L_0.8_, L_1.6_ and L_2.2_ are the top of atmosphere (TOA) radiances $\left[ {\frac{{W^{2} }}{{m^{2} m \cdot sr \cdot \mu m}}} \right]$, which were measured, for each pixel of the scene, at wavelengths around 0.8 µm (NIR), and 1.6 µm as well as 2.2 µm (SWIR), i.e. the spectral bands 5/6/7 for Landsat-8 OLI and 8A/11/12 for Sentinel-2 MSI. A pixel is classified as hotspot with strong thermal emission if NHI_SWNIR_ > 0 or as less intensive thermal emitting hotspot if NHI_SWIR_ > 0. In addition, we computed the total SWIR radiance of each detected thermal anomalies for each sensor. Furthermore, by combining the hotspots detected by Landsat-8 OLI and Sentinel-2 MSI, we estimated the total hotspot area. The main processing was performed using the Google Earth Engine Apps *NHI Tool for volcanoes* (version 1.4)^45^. In addition, we performed an offline processing of the Landsat-8 OLI and Sentinel-2 MSI scenes, for a more detailed analysis. We performed this analysis by running the algorithm without using the spectral test implemented within the NHI tool to remove artefacts associated to the multispectral misregistration of Sentinel-2 MSI imagery (see^45^).

Moreover, we measured the spatio-temporal evolution of the area of old Late’iki Island and New Late’iki Island prior, during and after the eruption period of Late’iki Volcano using the high spatial resolution Sentinel-2 (band combination 12/4/2) and Landsat-8 (band combination 7/4/2) data.

#### Thermal remote sensing satellite monitoring

For both VIIRS and MODIS hotspots, the volcanic radiant power (VRP) was derived using the well-established MIR-approach, where the measured heat flux is related to lava portions having a radiating temperature above 600 K^54^. For the VRP calculation of the VIIRS data the M13 band (spatial resolution 750 m) is used, due to the frequent hotspot pixel saturation in the co-located MIR I4 band. As mentioned in “Data”, the thermal hotspot pixel detection itself is performed using the 375 m spatial resolution I4 and I5 band data. A single pixel 750 m VRP retrieval is divided among the number of coincident 375 m hotspot pixels, with each sub-pixel receiving the same resulting value in Watts. As both sensors perform several overflights per day, the VRP of the overflight with the highest VRP value within each day was considered in the analysis.

#### SAR remote sensing satellite monitoring

Following processing steps were executed for TerraSAR-X SSC (Single-Look Slant Range Complex) and Sentinel-1 Single Look Complex (SLC) data: radiometric calibration to sigma nought, speckle filtering using the refined Lee speckle filter with a 7 × 7 pixel window^55^ and finally conversion from the radar imaging coordinates to a map projection (UTM/WGS1984 zone 1 south). Because New Late’iki was a newly born and short living island, no digital elevation model (DEM) was available.

Furthermore, polarimetric SAR processing was performed with the dual-pol Sentinel-1 data acquired over Late’iki Volcano. Thereby, after radiometric calibration and polarimetric speckle filtering using the refined Lee polarimetric speckle filter (with a kernel window of 7 × 7 pixels), which preserves the correlation between the different polarizations, the so-called entropy/alpha (H/α) polarimetric decomposition was performed. Based on physical assumptions, polarimetric decomposition procedures aim to separate different backscatter types^56^. As only dual-pol SAR data was available over the study site, we performed the dual-pol version of the H/α decomposition, which is based on the eigenvalues and eigenvectors of the coherency matrix^57^. The entropy H represents the heterogeneity of the scattering and ranges from 0 (indicating a dominant scatterer such as a corner reflector) to 1 (a random mixture of scattering mechanisms). The α angle describes the type of backscattering and ranges from surface scattering with low α values of ~ 0°, over volume scattering with α ~ 45°, to double-bouncing with α up to 90°. Next, based on the result of the H/α decomposition, the hereon based unsupervised Wishart classification was applied^58^. The initialization of the different clusters used in the Wishart classification is based on the following: The pixels of the SAR image are segmented into nine areas based on α and H^58^. The Wishart polarimetric classification procedure executes a Maximum Likelihood statistical segmentation of a polarimetric data set based on the multivariate complex Wishart probability density function. Three iterations were performed. Finally, result of the PolSAR classification was transformed to the aforementioned UTM map projection.

#### Seismic data

We investigated all seismic events listed in the three aforementioned global seismic catalogs (cf. “Seismic data”) for an area of 100 km radius around Late’iki Volcano and with a depth of < 100 km. We note that the closest seismic station is located on the Fiji Islands, around 770 km away from Late’iki Volcano. Due to the long distance of the seismic stations to Late’iki Volcano and because of other seismic events (earthquakes), it was not possible to detect clear evidence of seismic readings of Late’iki Volcano unrest (cf. “Joint analysis of all remote sensing data”).

## Supplementary Information

Supplementary Information.
