# Supplementary material for: The short life of the volcanic island New Late’iki (Tonga) analyzed by multi-sensor remote sensing data
Source: Sci Rep. 2020 Dec 18;10:22293. doi: 10.1038/s41598-020-79261-7 (PMC7749159; doi:10.1038/s41598-020-79261-7)
Supplement: Supplementary file 1 — Supplementary Information. [file 41598_2020_79261_MOESM1_ESM.pdf]

The short life of the volcanic island New Late'iki (Tonga) analyzed by multi-sensor remote sensing data

Simon Plank[1]*, Francesco Marchese[2], Nicola Genzano[3], Michael Nolde[1] and Sandro Martinis[1]

Supplementary information

Supplementary Table 1: Satellite imagery analyzed.

Acquisition date	Sensor	Acquisition date	Sensor
Sep. 1, 2019 – Feb. 29, 2020	MODIS	Nov. 3, 2019	Sentinel-1
	VIIRS	Nov. 9, 2019	Sentinel-2
Sep. 4, 2019	Sentinel-1	Nov. 10, 2019	Sentinel-1
Sep. 5, 2019	Sentinel-2	Nov. 14, 2019	Sentinel-2
Sep. 10, 2019	Sentinel-2	Nov. 15, 2019	Sentinel-1
Sep. 11, 2019	Sentinel-1	Nov. 19, 2019	Sentinel-2
Sep. 15, 2019	Sentinel-2	Nov. 22, 2019	Sentinel-1
Sep. 16, 2019	Sentinel-1	Nov. 24, 2019	Sentinel-2
Sep. 23, 2019	Sentinel-1	Nov. 27, 2019	Sentinel-1
Sep. 25, 2019	Sentinel-2	Dec. 2, 2019	TerraSAR-X[a)]
Sep. 28, 2019	Sentinel-1	Dec. 4, 2019	TerraSAR-X[b)]
Oct. 5, 2019	Sentinel-1	Dec. 9, 2019	Sentinel-2
	Sentinel-2	Dec. 14, 2019	Sentinel-2
Oct. 10, 2019	Sentinel-1	Dec. 24, 2019	Sentinel-2
	Sentinel-2	Dec. 29, 2019	Sentinel-2
Oct. 15, 2019	Sentinel-2	Jan. 3, 2020	Sentinel-2
Oct. 16, 2020	Landsat-8	Jan. 8, 2020	Sentinel-2
Oct. 17, 2019	Sentinel-1	Jan. 23, 2020	Sentinel-2
Oct. 20, 2019	Sentinel-2	Jan. 28, 2020	Sentinel-2
Oct. 22, 2019	Sentinel-1	Feb. 2, 2020	Sentinel-2
Oct. 29, 2019	Sentinel-1	Feb. 7, 2020	Sentinel-2
Oct. 30, 2019	Sentinel-2	Feb. 17, 2020	Sentinel-2

[a)] TerraSAR-X: SL; [b)] TerraSAR-X: HS

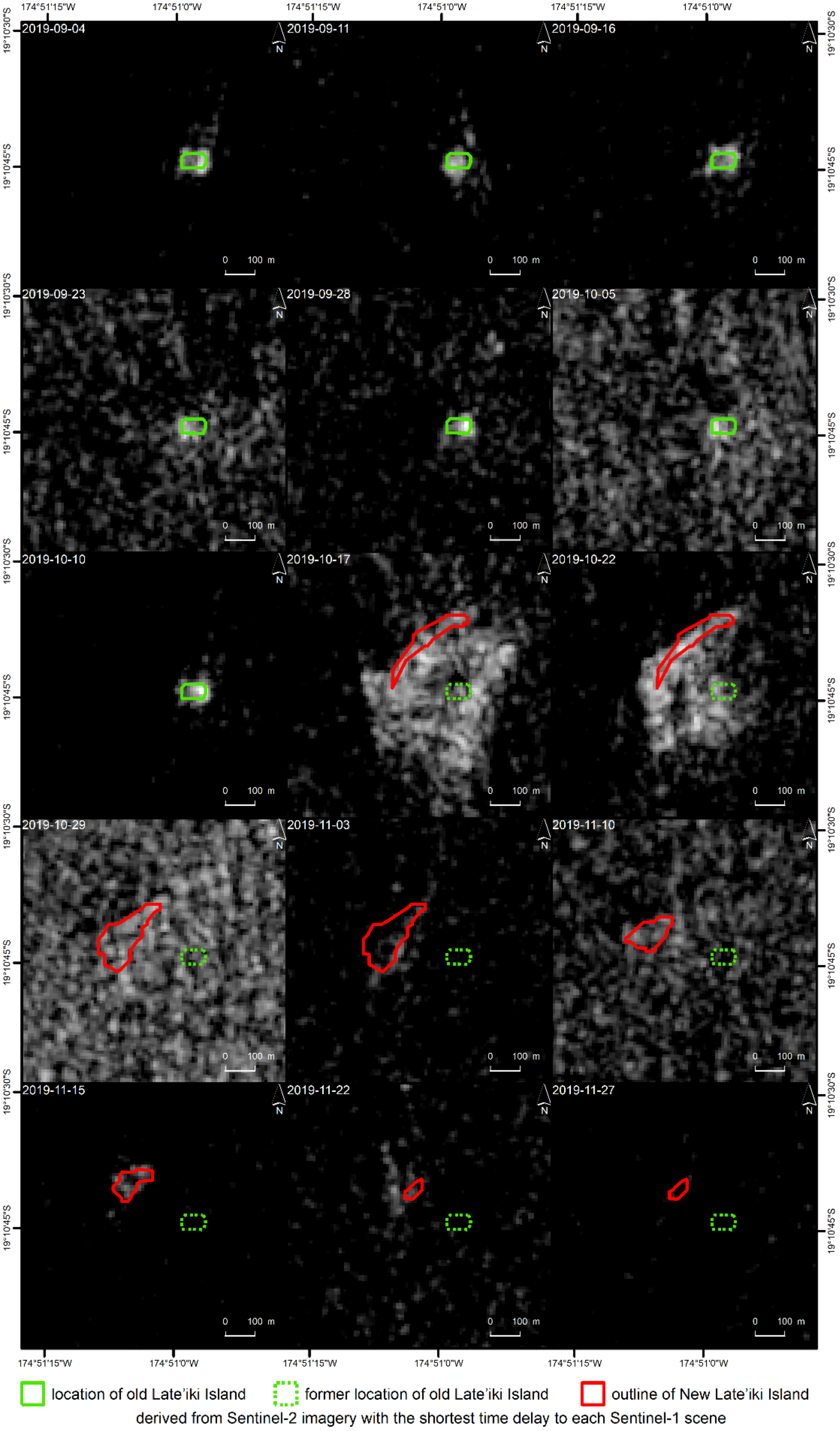

Supplementary Figure S1: Late'iki Volcano as observed by Sentinel-1 SAR VV-polarized imagery from 4 September 2019 to 27 November 2019. Background: Sentinel-1 Copernicus data (2019).

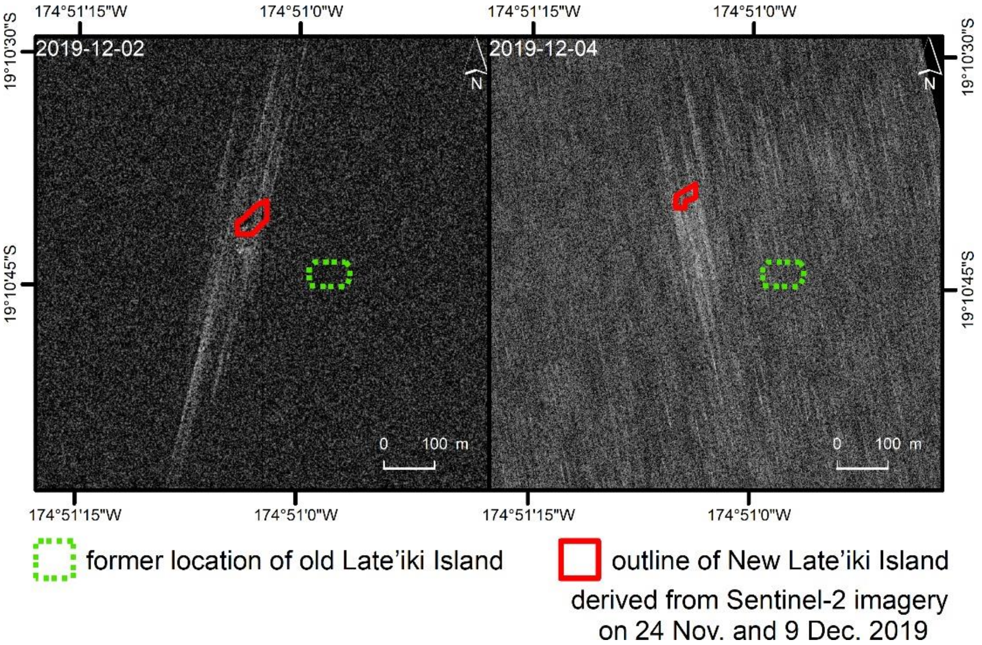

Supplementary Figure S2: The remains of New Late'iki Island as observed by TerraSAR-X on 2 December (SL mode) and on 4 December 2019 (HS mode), shortly before the entire island was eroded below the sea surface. Background: TerraSAR-X/TanDEM-X (DLR e.V. 2019).

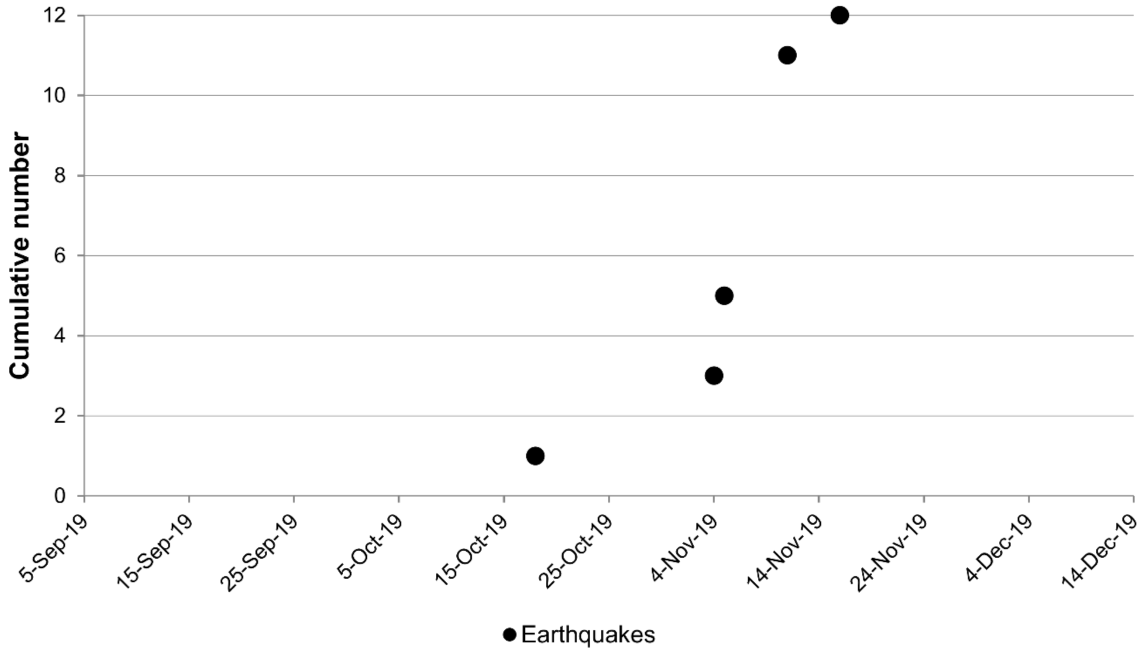

Supplementary Figure S3: Cumulative number of earthquakes for an area of 100 km radius around Late'iki Volcano and with a depth of < 100 km.
